# Clonality analysis of lymphoid proliferations using the BIOMED-2 clonality assays: a single institution experience

**DOI:** 10.2478/raon-2013-0072

**Published:** 2014-04-25

**Authors:** Ira Kokovic, Barbara Jezersek Novakovic, Petra Cerkovnik, Srdjan Novakovic

**Affiliations:** 1 Department of Molecular Diagnostics, Institute of Oncology Ljubljana, Ljubljana, Slovenia; 2 Department of Medical Oncology, Institute of Oncology Ljubljana, Ljubljana, Slovenia

**Keywords:** BIOMED-2, clonality analysis, lymphomas, IGH rearrangement, TCR rearrangement

## Abstract

**Background:**

Clonality determination in patients with lymphoproliferative disorders can improve the final diagnosis. The aim of our study was to evaluate the applicative value of standardized BIOMED-2 gene clonality assay protocols for the analysis of clonality of lymphocytes in a group of different lymphoid proliferations.

**Materials and methods.:**

With this purpose, 121 specimens from 91 patients with suspected lymphoproliferations submitted for routine diagnostics from January to December 2011 were retrospectively analyzed. According to the final diagnosis, our series comprised 32 cases of B-cell lymphomas, 38 cases of non-Hodgkin’s T-cell lymphomas and 51 cases of reactive lymphoid proliferations. Clonality testing was performed using the BIOMED-2 clonality assays.

**Results:**

The determined sensitivity of the TCR assay was 91.9%, while the sensitivity of the IGH assay was 74.2%. The determined specificity of the IGH assay was 73.3% in the group of lymphomas and 87.2% in the group of reactive lesions. The determined specificity of the TCR assay was 62.5% in the group of lymphomas and 54.3% in the group of reactive lesions.

**Conclusions:**

In the present study, we confirmed the utility of standardized BIOMED-2 clonality assays for the detection of clonality in a routine diagnostical setting of non-Hodgkin’s lymphomas. Reactions for the detection of the complete *IGH* rearrangements and reactions for the detection of the *TCR* rearrangements are a good choice for clonality testing of a wide range of lymphoid proliferations and specimen types while the reactions for the detection of incomplete *IGH* rearrangements have not shown any additional diagnostic value.

## Introduction

In the majority of patients with suspected lymphoproliferations (LP), the diagnosis can be done by histomorphology or cytomorphology, supplemented with immunohistochemistry or flow cytometric immunophenotyping.^1^ However, in 5–15% of patients the morphological and immunophenotypic features are not typical and the diagnosis is more complicated. In such cases, molecular clonality analysis of lymphocyte populations may contribute to the diagnosis.^1^,^2^ Clonality analysis of lymphoid cells using the polymerase chain reaction (PCR) to amplify V-(D)-J junctional regions of immunoglobulin (*Ig*) and T-cell receptor (*TCR*) genes enables the discrimination between polyclonal, reactive processes and monoclonal, malignant tumors.^1^–^3^ Since the introduction of PCR-based as-says in the early nineties, different strategies with different primer sets have been developed and used for determination of B and T-cell clonality.^4^–^12^ However, many of those PCR-based clonality as-says were designed to cover a limited number of possible *Ig* and *TCR* gene rearrangements, resulting in false negative results.^3^,^13^

A comprehensive work of the European BIOMED-2 collaborative study group (now called the EuroClonality consortium) led to new standardized PCR protocols with multiple primer sets for the clonality analysis of both *Ig* and *TCR* gene rearrangements in a diagnostic setting.^14^ In initial studies, novel BIOMED-2 multiplex PCR protocols were evaluated on large series of B-cell and T-cell malignancies, and histomorphologically reactive lesions.^15^–^17^ Based on their conclusions, the BIOMED-2 clonality assays were declared as highly sensitive, specific and reproducible, and thus reliable for detection of clonality in lymphoid malignancies.^15^–^17^ The guidelines for use of these assays in the routine clonality testing have been proposed.^3^ Recommendations for correct interpretation and potential pitfalls in the Ig/TCR clonality testing were also presented.^18^,^19^ Over the past decade, a number of studies have reported the successful application of the BIOMED-2 clonality assays in a diagnostic setting.^20^–^38^ Some studies have evaluated subsets of BIOMED-2 primers for clonality analysis in selected specimen types - fixed and decalcified bone marrow biopsies^23^, archival skin biopsy samples^24^, formalin-fixed and paraffin-embedded specimens^26^,^29^ and fine needle aspiration biopsies.^27^ The others applied BIOMED-2 assays to different disease sub-categories – B-cell precursor acute lymphoblastic leukemia^21^, classical Hodgkin’s lymphoma^26^, follicular lymphoma^28^,^29^, cutaneous lymphoproliferations^30^, anaplastic large cell lymphoma and peripheral T-cell lymphomas^32^, Mycosis fungoides and inflammatory dermatoses^33^, polymorphous lymphoproliferative disorders in individuals with immunodeficiency conditions^34^ and granulomatous disorders.^35^ Thus, the BIOMED-2 clonality assays have become the world standard for PCR-based Ig/TCR clonality testing.^39^ Moreover, the EuroClonality consortium recently developed a uniform reporting system for describing results and conclusions of Ig/TCR clonality assays.^39^

The aim of our retrospective study was to evaluate the applicative value of standardized BIOMED-2 gene clonality assay protocols for the analysis of clonality of lymphocytes on a series of various diagnostic specimens (fresh and formalin-fixed) from Slovenian patients with different lymphoid proliferations.

## Materials and methods

### Study group

One hundred and twenty-one specimens from 91 patients with suspected non-Hodgkin’s lymphoma submitted for routine diagnostics from January to December 2011 were analyzed. Among diagnostic samples, bone marrow (BM) aspirates predominated (51), followed by formalin-fixed, paraffin-embedded tissue specimens (FFPE) (31) and fine-needle aspiration specimens (FNA) (31). A minority of specimens consisted of cerebrospinal fluid (1), pleural fluid (4), imprint cytology of lymph node (1) and ascites (2). All specimens were subjected to cyto/histomorphological and immunophenotyping examination as well as to molecular clonality analysis of lymphocyte populations during routine diagnostic assessment.

### DNA isolation

DNA from FFPE tissue specimens was isolated using the QIAamp FFPE tissue kit (Qiagen GmbH, Hilden, Germany). DNA from other types of specimens was isolated using High Pure PCR Template Preparation kit (Roche Applied Science, Penzberg, Germany) according to the manufacturers’ protocols. The concentration and the purity of DNA (A_260nm_/A_280nm_) were determined using the Nanodrop spectrophotometer (ThermoScientific, Wilmington, USA).

### Clonality analysis

Clonality analysis of lymphoid cells was performed using the BIOMED-2 clonality assays – ABI Fluorescence Detection (IdentiClone, *InVivo* Scribe Technologies, San Diego, CA, USA) according to the manufacturer’s instructions. B-cell clonality was assessed using the IdentiClone IGH Gene clonality assay for detection of clonal rearrangements in the immunoglobulin heavy chain gene (*IGH*). The T-cell clonality was assessed using the TCRB+TCRG Gene Clonality Assay for detection of clonal rearrangements in the T-cell receptor β chain gene (*TCRB*) and the T-cell receptor γ chain gene (*TCRG*).

The DNA quality was checked for all samples using the control gene PCR (Specimen Control Size Ladder master mix). The DNA was considered of adequate quality if amplified products of ≥400 bp were obtained in a control PCR, except for the DNA from FFPE tissue, which was considered acceptable if amplified products of ≥300 bp were obtained.

The *IGH* clonality was evaluated with five different IGH multiplex PCR reactions, three reactions for detection of the complete rearrangements (V_H_-J_H_) and two reactions for detection of the incomplete rearrangements in the *IGH* gene (D_H_–J_H_) (V – variable, D – diversity, J – joining gene segments, respectively). The *TCR* clonality was evaluated with three TCRB and two TCRG multiplex PCR reactions. In case of doubtful results, the as-says were repeated. Each run included monoclonal and polyclonal control DNAs for particular primer master mix, supplied with each BIOMED-2 clonality assay, and a contamination control (no template DNA in a reaction).

The fluorescently labeled PCR products were detected by capillary gel electrophoresis using the ABI 3500 Genetic Analyzer (Applied Biosystems, Foster City, CA, USA) and analyzed by fragment analysis. Amplified products from diagnostic samples were interpreted according to the manufacturer’s instructions. Samples that failed to amplify following repeated testing were reported as “not detected” (*i.e*. clonality could not be detected due to insufficient quality or quantity of DNA for analysis).

### Final diagnosis

The final diagnosis of each lymphoproliferation was set upon careful examination of all available information. Malignant lymphomas were classified according to the WHO Classification of Tumours of Hematopoietic and Lymphoid Tissues.^40^,^41^

### Sensitivity and specificity

To determine the sensitivity and the specificity of IGH/TCR clonality assays we compared the results of molecular testing with the final diagnosis of each lymphoproliferation. The sensitivity of each clonality assay was calculated using the following equation: TP/(TP+FN), in which TP represents the number of true positives and FN the number of false negatives. The specificity of each assay was calculated using the equation TN/(TN+FP), in which TN represents the number of true negatives and FP the number of false positives. The specificities of IGH and TCR assays were calculated separately for T/B-cell lymphomas and for reactive lymphoproliferations.

## Results

In the period from January to December 2011 we have analyzed 121 specimens from 91 patients (96 specimens were analyzed for *IGH* and 119 specimens for *TCR* clonality). Of these, 84 specimens were analyzed for both – B and T-cell clonality.

**FIGURE 1.**
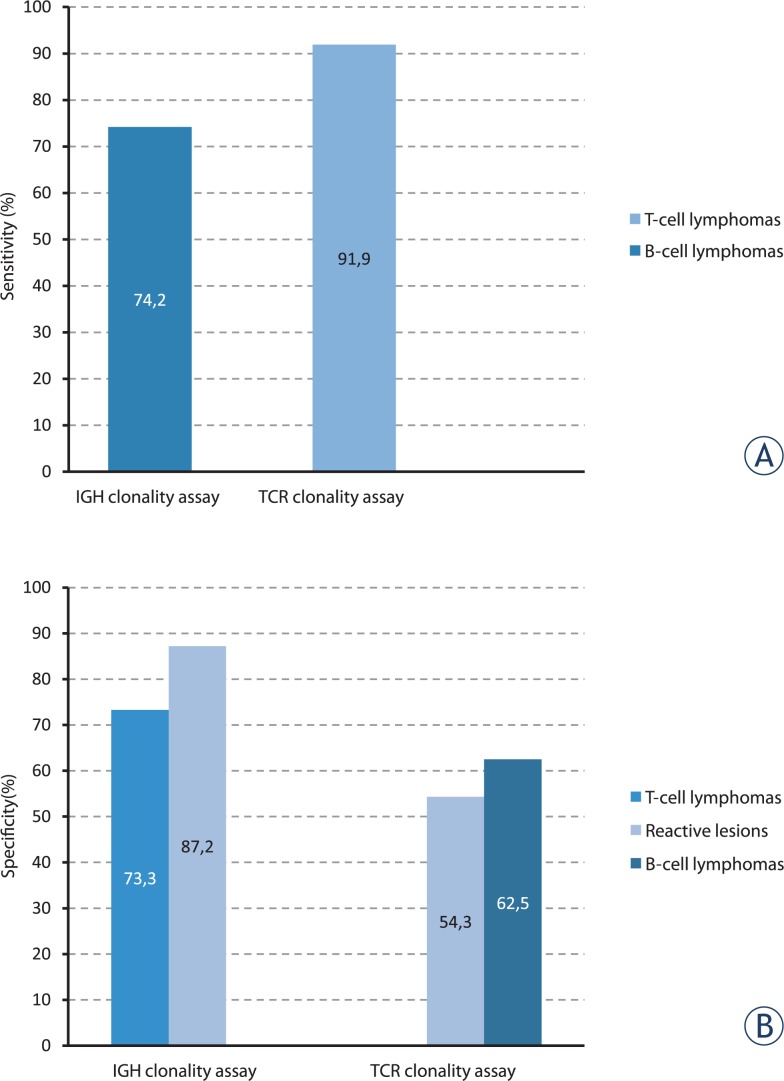
Sensitivity and specificity of the BIOMED-2 clonality assays determined in a group of T-cell lymphomas, B-cell lymphomas and reactive lesions. To determine the sensitivity and the specificity of IGH/TCR clonality assays the results of molecular testing were compared with the final diagnosis of each lymphoproliferation. The sensitivity of each clonality assay was calculated using the equation TP/(TP+FN); TP (true positives) – monoclonal (M) and “monoclonal in a polyclonal background” (M/P) results of the IGH clonality assay in a group of B-cell lymphomas, and M and M/P results of the TCR clonality assay in a group of T-cell lymphomas; FN (false negatives) – polyclonal (P) results of the IGH clonality assay in a group of B-cell lymphomas and P results of the TCR clonality assay in a group of T-cell lymphomas. The specificity of the IGH clonality assay was determined separately for T-cell lymphomas and for reactive lesions. Similarly, the specificity of the TCR clonality assay was determined separately for B-cell lymphomas and for reactive lesions. The specificity of each assay was calculated using the equation TN/(TN+FP); TN (true negatives) – P results of the IGH clonality assay in a group of T-cell lymphomas or in reactive lesions, and P results of the TCR clonality assay in a group of B-cell lymphomas or in reactive lesions; FP (false positives) – M and M/P results of the IGH clonality assay in a group of T-cell lymphomas or reactive lesions, and M and M/P results of the TCR clonality assay in a group of B-cell lymphomas or in reactive lesions.

According to the final diagnosis, our diagnostic series comprised 32 cases of B-cell lymphomas, 38 cases of non-Hodgkin’s T-cell lymphomas and 51 cases of reactive lymphoid proliferations. The results of clonality analysis using BIOMED-2 clonality assays are shown in Table 1. The calculated sensitivities and specificities of BIOMED-2 clonality assays after fragment analysis of amplified products are shown in Figure 1.

### The BIOMED-2 IGH clonality assay

It was performed in 96 specimens (Table 2). In total, monoclonal *IGH* rearrangements corresponding to monoclonal B-cell proliferations were detected in 32 cases (33.3%). Polyclonal *IGH* rearrangements corresponding to polyclonal B-cell proliferations were detected in 60 cases (62.5%). One case of B-cell non-Hodgkin’s lymphoma (B-NHL) was concluded as borderline (“monoclonal in a polyclonal background”). In three cases the B-cell clonality could not be determined either because of an insufficient number of B-cells in the specimen or due to the poor quality of the isolated DNA (in one case of histopathologically confirmed B-NHL and in two reactive specimens). Among B-cell lymphomas, 22 of 32 analyzed cases were monoclonal, representing “true positives” and 8 cases were polyclonal, representing “false negatives”. Polyclonal *IGH* rearrangements in a group of B-cell lymphomas were detected in follicular lymphoma (FL) (3 cases), marginal zone B-cell lymphoma (MZL) (3 cases), diffuse large B-cell lymphoma (DLBCL) (1 case) and B-cell lymphoma with features intermediate between the DLBCL and Burkitt’s lymphoma (BL) (1 case).

In the group of T-cell lymphomas, 11 of 15 analyzed cases were polyclonal by the IGH assay, representing “true negatives” and 4 cases were monoclonal, representing “false positives” - a case of T-cell lymphoma with monoclonal B-cell population and 3 specimens from a patient with angioimmunoblastic T-cell lymphoma (AITL) (2 FNAs and one BM aspiration). Among 51 specimens of reactive lymphoid proliferations (R), monoclonal *IGH* rearrangements were detected in 6 cases (“false positives”) and polyclonal *IGH* rearrangements were detected in 41 cases (“true negatives”).

### Incomplete rearrangements in the *IGH* gene

Besides complete rearrangements in the *IGH* gene we also detected incomplete rearrangements (D_H_J_H_) in a few cases. In total, we detected 8 incomplete rearrangements in 96 specimens of LP analyzed by the IGH assay (8.3%). Four were detected by the IGH-D reaction containing D_H1-6_ and J_H_ primers (one in case of T-NHL and 3 in reactive lymphoid proliferations) and four by the IGH-E reaction containing D_H7_ and J_H_ primers (one in case of FL, one in case of T-NHL and two in reactive specimens).

### The BIOMED-2 TCR clonality assay

It was performed in 109 specimens (Table 3). In total, by using the BIOMED-2 TCR clonality assay, the T-cells with monoclonal rearrangements were detected in 61 specimens, while T-cells with polyclonal rearrangements were detected in 43 cases. Borderline results (“monoclonal in a polyclonal background”) were obtained in 3 specimens (two BM specimens taken for staging/follow-up of T-cell non-Hodgkin’s lymphomas (T-NHL) and one FNA specimen with reactive lymphoproliferation). The T-cell clonality was not assessed in 2 specimens due to fragmented DNA (in one case of T-NHL and in one case of B-NHL). In a group of primary T-cell lymphomas, monoclonal *TCRG* and/or *TCRB* rearrangements were detected in 32 of 38 analyzed cases, representing “true positives”. Three of 38 T-NHL cases were polyclonal by the TCR assay, representing “false negatives”. Among T-NHLs polyclonal *TCR* rearrangements were detected in 2 cases of peripheral T-cell lymphoma, otherwise unspecified (PTCL-U) and in one case of peripheral T-cell lymphoma, cutaneous.

In the group of B-NHLs monoclonal rearrangements in *TCR* genes were detected in 9 of 25 analyzed cases (“false positives”). Fifteen cases of B-NHL had polyclonal rearrangements in *TCR* genes, as expected (“true negatives”). Among reactive lymphoid proliferations monoclonal *TCR* rearrangements were detected in 20 cases (“false positives”) and polyclonal TCR rearrangements were detected in 25 cases (“true negatives”).

## Discussion

The aim of this study was to evaluate the application value of BIOMED-2 clonality assays for analysis of different lymphoid proliferations in the diagnostic setting. With this purpose, we analyzed 121 specimens from 91 patients with suspected lymphoproliferations. The clonality testing was performed using the BIOMED-2 clonality as-says according to the guidelines proposed by the European BIOMED-2/EuroClonality group and the results of clonality testing were interpreted in the context of the final diagnosis.

### BIOMED-2 IGH assay

The sensitivity as well as the specificity of the IGH assay in our diagnostic series of NHL cases was lower than expected. The sensitivity of the IGH assay in our diagnostic series was 74.2%, while the BIOMED-2/EuroClonality group reported the sensitivity of 91.0%, ranging from 85–100% depending on the disease category.^3^ Similarly, the determined specificity of the IGH assay was 73.3% in our NHL cases and 87.2% in the group of reactive specimens, again lower than reported in the BIOMED-2 study, where the overall specificity of the IGH clonality assay in T-NHL was almost 91.0%.^16^

The lower sensitivity in our series may be related to a smaller number of included B-NHL cases (only 32) as well as to a rather high percentage of germinal center (GC)/post-GC lymphomas which predominated in our group of B-NHLs (28 of 32, including 14 of 15 BM aspirates taken for staging/ follow-up) (Table 2). It is namely well known that somatic mutations in the *IGH* gene are frequent in GC/post-GC B-cell lymphomas, especially in FL^42^, which contributes to a lower monoclonality rate.^3^,^13^,^28^,^29^ Indeed, all B-NHL cases with polyclonal *IGH* rearrangements in our study (8/32) were from the group of GC/post-GC lymphomas, including FL (3), marginal zone B-cell lymphoma (3), DLBCL (1) and B-cell lymphoma with features intermediate between the DLBCL and Burkitt (1).

The lower overall specificity of the IGH clonality assay in our series of NHL cases can be explained by the T-NHL entities in which monoclonal *IGH* rearrangements were detected: a case of T-cell lymphoma with monoclonal B-cell population and 3 specimens from a patient with angioimmunoblastic T-cell lymphoma (AITL). This is in concordance with the results from the BIOMED-2 study in which monoclonal *IGH* rearrangements were mostly detected in AITL (in 30.0% of cases)^16^ and also with the results of other studies where the presence of monoclonal *IGH* rearrangements in AITL was reported in 17.6% of cases.^38^ The fore mentioned studies have shown that monoclonal *IGH* gene rearrangements occur in 5–10% of all T-cell malignancies and represent the so-called cross-rearrangements, which sometimes occur in more immature lymphoid cells.^3^,^16^

Unlike in the T-NHL cases, the specificity of the IGH assay in our series of reactive lesions (87.2%) is comparable to the results from the BIOMED-2 study.^17^ In our study, clearly polyclonal IGH products were determined in 41 of 49 reactive specimens (Table 1). In 6 cases, the monoclonal IGH products were detected - three BM aspirates taken for staging/follow-up of B-NHLs, two FFPE specimens suspective of lymphoma and one FNA specimen suspective of granulomatous lymphadenitis. A further pathological review of these cases did not show any cells suspective of B-cell lymphoma and were concluded as reactive lymphoproliferations. As previously stated, monoclonal results in reactive specimens must be interpreted with caution in the context of all clinical, morphological and immunophenotyping data.^1^,^3^ Concerning this, in all 6 cases a close follow-up and the re-sampling were recommended.

An important aspect of our study was the evaluation of the utility of reactions for the detection of incomplete rearrangements in the *IGH* gene, which can be detected in ∼30% of B-cell malignancies.^3^ In contrast to our expectations, among B-NHL cases (32) we detected only one incomplete rearrangement by the IGH-E reaction (3.1%) - it was in case of FL, which was polyclonal in reactions targeting the complete rearrangements. Since histopathological diagnosis of this case was difficult, the detection of incomplete rearrangement might have served as an additional evidence of malignant process. The follow-up of this patient was strongly recommended. Considering the low frequency of incomplete rearrangements in all analyzed specimens of LP and the fact that only one additional monoclonal result was obtained in the group of B-NHLs we concluded that IGH-D and IGH-E reactions did not have any additional diagnostic value. Our results are in agreement with the study on 118 FFPE specimens from patients with FL, in which also no additional monoclonal results were detected with reactions targeting incomplete rearrangements (IGH-D and IGH-E).^29^

### BIOMED-2 TCR clonality assay

The overall sensitivity of the TCR clonality assay in our diagnostic series was 91.9% which is in agreement with the reported sensitivity of the BIOMED-2/EuroClonality group (91.0%).^16^ The TCRB clonality assay showed a higher analytical detection rate (76.3%) than the TCRG clonality assay (63.2%), which is in agreement with the results of the BIOMED-2 study.^16^

As we have previously shown for the IGH assay, the specificity of the TCR clonality assay in our study was lower than described by founders of the protocol.^3^,^14^ The determined specificity was 62.5% in the group of B-NHLs and 54.3% in the group of reactive lymphoid proliferations. However, the detection of monoclonal rearrangements in *TCR* genes in our series of B-NHL cases and reactive lesions is consistent with the findings from other studies, which have shown that the co-existing small T-cell populations are frequently present in both, B-cell malignancies and reactive specimens.^15^,^17^ The rearrangements in *TCR* genes occur in 10–20% of B-cell malignancies and are generally reported to be found in a single *TCR* locus.^3^,^15^ In contrast, we have detected multiple monoclonal results in TCRB and TCRG reactions in 5 cases of B-NHL. Interestingly, 4 of 5 specimens with monoclonal rearrangements in both *TCRB* and *TCRG* loci were BM aspirates taken for staging: MZL (2), FL (1) and DLBCL (1) (results not shown). One FNA specimen of initially suspected B-NHL with monoclonal rearrangements in both *IGH* and *TCR* genes was later reclassified as the T-cell lymphoma with a monoclonal B-cell population after an additional pathological examination of pleural fluid obtained from the same patient.

The unexpectedly high frequency (43.5%) of monoclonal *TCR* rearrangements in reactive specimens in our study is difficult to explain (Table 3). It is well known that the *TcR*γ gene has a restricted germline repertoire and a limited junctional diversity at the rearranged Vγ-Jγ region, and thus theoretically carries the risk of pseudoclonal products in samples containing small numbers of T-cells.^14^ There is a possibility of detecting pseudoclonal products by the TCRG clonality assay in 4 of 20 cases, which were monoclonal by the TCRG clonality assay and polyclonal by the TCRB clonality assay. However, in 10 of 20 cases monoclonal *TCR* rearrangements were detected by both assays and can hardly be interpreted as pseudoclonal. It is of note that monoclonal *TCR* rearrangements in our cases with the final diagnosis of reactive lesions were mostly detected in BM aspirates (14/20 BM aspirates) (Table 3). The majority of BM aspirates with monoclonal *TCR* rearrangements (11/14) were taken for staging, all from patients initially suspective of having B-cell malignancies. Three specimens were taken for the assessment of minimal residual disease (MRD), the first from a patient with AITL, the second from a patient with plasmablastic lymphoma and the third from a patient with FL. All three were concluded as reactive BM specimens according to morphological and immunophenotyping data. However, it should be postulated that monoclonal *TCR* rearrangements might not always be clinically significant, since monoclonal T-cell populations can be detected in peripheral blood of the elderly, in patients with autoimmune diseases and in patients with viral infection.^43^–^45^

In the present study we confirmed the application value of standardized BIOMED-2 clonality assays for the detection of clonality in a routine diagnostic setting of non-Hodgkin’s lymphomas. Our conclusions are that (i) three reactions for detection of complete *IGH* rearrangements and five reactions for detection of *TCR* rearrangements (targeting both *TCRB* and *TCRG* genes) are a good choice for the clonality testing in these lymphomas; (ii) reactions for the detection of incomplete *IGH* rearrangements have not shown any additional diagnostic value in our hands; (iii) due to the lower sensitivity of the IGH clonality assay in our study, we should consider the introduction of the IGK clonality assay as an additional clonality test, especially in cases of GC/post-GC B-cell malignancies; (iv) detection of monoclonal rearrangements in both *IGH* and *TCR* genes must be interpreted with caution and in the context of all clinical, morphological and immunophenotyping data, as discussed elsewhere.

We are aware that our conclusions derive from a rather small diagnostic series of 121 specimens of different lymphoid proliferations with only 32 cases of confirmed B-NHLs and 38 cases of confirmed T-NHLs. Certainly, the evaluation of larger series of B and T-cell lymphomas and reactive lesions needs to be done for firmer conclusions. Yet, we believe that our results might be useful for other laboratories aiming to introduce the standardized BIOMED-2 clonality assays in a routine laboratory practice.

## Figures and Tables

**TABLE 1.** Results of clonality analysis using BIOMED-2 clonality assays in suspected lymphoid proliferations

**Final diagnosis (n)**	**IGH clonality**					**TCR clonality**				
	**M (n)**	**P (n)**	**M/P (n)**	**ND (n)**	**NP (n)**	**M (n)**	**P (n)**	**M/P (n)**	**ND (n)**	**NP (n)**
**B-NHL (32)**	22	8	1	1	0	9	15	0	1	7
**T-NHL (38)**	4	11	0	0	23	32	3	2	1	0
**Reactive (51)**	6	41	0	2	2	20	25	1	0	5
**TOTAL (121)**	**32**	**60**	**1**	**3**	**25**	**61**	**43**	**3**	**2**	**12**

B-NHL = B-cell non-Hodgkin’s lymphoma; T-NHL = T-cell non-Hodgkin’s lymphoma; M = monoclonal; P = polyclonal; M/P =monoclonal in a polyclonal background; ND = not detected; NP = not performed

**TABLE 2.** Detection of monoclonal *IGH* gene rearrangements in 96 specimens of lymphoid proliferations (LP)

**Diagnosis**	**No. of monoclonal / No. of tested specimens (%)**
B-NHL - Primary tumor	11/17 (64.7)
MALT lymphoma	1/2 (50.0)
Follicular lymphoma	3/4 (75.0)
Diffuse large B-cell lymphoma	1/2(50.0)
Marginal zone B-cell lymphoma	2/4 (50.0)
Lymphoplasmacytic lymphoma	2/2 (100.0)
B-NHL, unclassified	2/3 (66.7)
B-NHL - Staging/follow-up BM^a^	11/15 (73.3)

Total B-NHL	22/32 (68.8)
T-NHL	4/15 (26.7)
Reactive specimens	6/49 (12.2)

TOTAL LP	32/96 (33.3)

B-NHL = B-cell non-Hodgkin’s lymphoma; T-NHL = T-cell non-Hodgkin’s lymphoma; MALT lymphoma = extranodal marginal zone lymphoma of mucosa-associated tissue; LP = lymphoid proliferation

a Bone marrow (BM) aspirates were taken from different patients with marginal zone B-cell lymphoma (4), diffuse large B-cell lymphoma (4), follicular lymphoma (3), MALT lymphoma (1), mantle cell lymphoma (1), plasmablastic lymphoma (1) and lymphoplasmacytic lymphoma (1).

**TABLE 3.** Detection of monoclonal *TCR* gene rearrangements in 109 specimens of lymphoid proliferations (LP)

**Diagnosis**	**No. of monoclonal / No. of tested specimens (%)**
T-NHL – Primary tumor	22/26 (84.6)
Peripheral T-cell lymphoma, unspecified	12/15 (80.0)
Peripheral T-cell lymphoma, cutaneous	1/2 (50.0)
Angioimmunoblastic T-cell lymphoma	8/8 (100.0)
Mycosis fungoides/Sezary syndrome	1/1 (100.0)
T-NHL - Staging/follow-up BM^a^	10/12 (83.3)

Total T-NHL	32/38 (84.2)
B-NHL	9/25 (36.0)
Reactive specimens^b^	20/46 (43.5)

TOTAL LP	61/109 (56.0)

B-NHL = B-cell non-Hodgkin’s lymphoma; T-NHL = T-cell non-Hodgkin’s lymphoma

a Bone marrow (BM) aspirates were taken from patients with peripheral T-cell lymphoma, unspecified (4), angioimmunoblastic T-cell lymphoma (3), T-lymphoblastic lymphoma (3), NK/T-cell lymphoma (1) and T-cell acute lymphoblastic leukaemia (1).

b Reactive specimens included 20 BM aspirates, 17 FNA specimens of lymph nodes and 9 FFPE specimens.
